# Prevalence and Characteristics of Autism Spectrum Disorder Among Children Aged 4 Years — Early Autism and Developmental Disabilities Monitoring Network, Seven Sites, United States, 2010, 2012, and 2014

**DOI:** 10.15585/mmwr.ss6802a1

**Published:** 2019-04-12

**Authors:** Deborah L. Christensen, Matthew J. Maenner, Deborah Bilder, John N. Constantino, Julie Daniels, Maureen S. Durkin, Robert T. Fitzgerald, Margaret Kurzius-Spencer, Sydney D. Pettygrove, Cordelia Robinson, Josephine Shenouda, Tiffany White, Walter Zahorodny, Karen Pazol, Patricia Dietz

**Affiliations:** 1 Division of Congenital and Developmental Disorders, National Center on Birth Defects and Developmental Disabilities, CDC; 2 University of Utah, Salt Lake City; 3 Washington University in St. Louis, Missouri; 4 University of North Carolina, Chapel Hill; 5 University of Wisconsin, Madison; 6 University of Arizona, Tucson; 7 University of Colorado School of Medicine, Aurora; 8 Rutgers New Jersey Medical School, Newark; 9 Colorado Department of Public Health and Environment, Denver

## Abstract

**Problem/Condition:**

Autism spectrum disorder (ASD) is estimated to affect up to 3% of children in the United States. Public health surveillance for ASD among children aged 4 years provides information about trends in prevalence, characteristics of children with ASD, and progress made toward decreasing the age of identification of ASD so that evidence-based interventions can begin as early as possible.

**Period Covered:**

2010, 2012, and 2014.

**Description of System:**

The Early Autism and Developmental Disabilities Monitoring (Early ADDM) Network is an active surveillance system that provides biennial estimates of the prevalence and characteristics of ASD among children aged 4 years whose parents or guardians lived within designated sites. During surveillance years 2010, 2012, or 2014, data were collected in seven sites: Arizona, Colorado, Missouri, New Jersey, North Carolina, Utah, and Wisconsin. The Early ADDM Network is a subset of the broader ADDM Network (which included 13 total sites over the same period) that has been conducting ASD surveillance among children aged 8 years since 2000. Each Early ADDM site covers a smaller geographic area than the broader ADDM Network. Early ADDM ASD surveillance is conducted in two phases using the same methods and project staff members as the ADDM Network. The first phase consists of reviewing and abstracting data from children’s records, including comprehensive evaluations performed by community professionals. Sources for these evaluations include general pediatric health clinics and specialized programs for children with developmental disabilities. In addition, special education records (for children aged ≥3 years) were reviewed for Arizona, Colorado, New Jersey, North Carolina, and Utah, and early intervention records (for children aged 0 to <3 years) were reviewed for New Jersey, North Carolina, Utah, and Wisconsin; in Wisconsin, early intervention records were reviewed for 2014 only. The second phase involves a review of the abstracted evaluations by trained clinicians using a standardized case definition and method. A child is considered to meet the surveillance case definition for ASD if one or more comprehensive evaluations of that child completed by a qualified professional describes behaviors consistent with the *Diagnostic and Statistical Manual of Mental Disorders, 4th Edition, Text Revision* (DSM-IV-TR) diagnostic criteria for any of the following conditions: autistic disorder, pervasive developmental disorder–not otherwise specified (PDD-NOS, including atypical autism), or Asperger disorder (2010, 2012, and 2014). For 2014 only, prevalence estimates based on surveillance case definitions according to DSM-IV-TR and the *Diagnostic and Statistical Manual of Mental Disorders, Fifth Edition* (DSM-5) were compared. This report provides estimates of overall ASD prevalence and prevalence by sex and race/ethnicity; characteristics of children aged 4 years with ASD, including age at first developmental evaluation, age at ASD diagnosis, and cognitive function; and trends in ASD prevalence and characteristics among Early ADDM sites with data for all 3 surveillance years (2010, 2012, and 2014), including comparisons with children aged 8 years living in the same geographic area. Analyses of time trends in ASD prevalence are restricted to the three sites that contributed data for all 3 surveillance years with consistent data sources (Arizona, Missouri, and New Jersey).

**Results:**

The overall ASD prevalence was 13.4 per 1,000 children aged 4 years in 2010, 15.3 in 2012, and 17.0 in 2014 for Early ADDM sites with data for the specific years. ASD prevalence was determined using a surveillance case definition based on DSM-IV-TR. Within each surveillance year, ASD prevalence among children aged 4 years varied across surveillance sites and was lowest each year for Missouri (8.5, 8.1, and 9.6 per 1,000, for 2010, 2012, and 2014, respectively) and highest each year for New Jersey (19.7, 22.1, and 28.4 per 1,000, for the same years, respectively). Aggregated prevalence estimates were higher for sites that reviewed education and health care records than for sites that reviewed only health care records. Among all participating sites and years, ASD prevalence among children aged 4 years was consistently higher among boys than girls; prevalence ratios ranged from 2.6 (Arizona and Wisconsin in 2010) to 5.2 boys per one girl (Colorado in 2014). In 2010, ASD prevalence was higher among non-Hispanic white children than among Hispanic children in Arizona and non-Hispanic black children in Missouri; no other differences were observed by race/ethnicity. Among four sites with ≥60% data on cognitive test scores (Arizona, New Jersey, North Carolina, and Utah), the frequency of co-occurring intellectual disabilities was significantly higher among children aged 4 years than among those aged 8 years for each site in each surveillance year except Arizona in 2010. The percentage of children with ASD who had a first evaluation by age 36 months ranged from 48.8% in Missouri in 2012 to 88.9% in Wisconsin in 2014. The percentage of children with a previous ASD diagnosis from a community provider varied by site, ranging from 43.0% for Arizona in 2012 to 86.5% for Missouri in 2012. The median age at earliest known ASD diagnosis varied from 28 months in North Carolina in 2014 to 39.0 months in Missouri and Wisconsin in 2012. In 2014, the ASD prevalence based on the DSM-IV-TR case definition was 20% higher than the prevalence based on the DSM-5 (17.0 versus 14.1 per 1,000, respectively).

Trends in ASD prevalence and characteristics among children aged 4 years during the study period were assessed for the three sites with data for all 3 years and consistent data sources (Arizona, Missouri, and New Jersey) using the DSM-IV-TR case definition; prevalence was higher in 2014 than in 2010 among children aged 4 years in New Jersey and was stable in Arizona and Missouri. In Missouri, ASD prevalence was higher among children aged 8 years than among children aged 4 years. The percentage of children with ASD who had a comprehensive evaluation by age 36 months was stable in Arizona and Missouri and decreased in New Jersey. In the three sites, no change occurred in the age at earliest known ASD diagnosis during 2010–2014.

**Interpretation:**

The findings suggest that ASD prevalence among children aged 4 years was higher in 2014 than in 2010 in one site and remained stable in others. Among children with ASD, the frequency of cognitive impairment was higher among children aged 4 years than among those aged 8 years and suggests that surveillance at age 4 years might more often include children with more severe symptoms or those with co-occurring conditions such as intellectual disability. In the sites with data for all years and consistent data sources, no change in the age at earliest known ASD diagnosis was found, and children received their first developmental evaluation at the same or a later age in 2014 compared with 2010. Delays in the initiation of a first developmental evaluation might adversely affect children by delaying access to treatment and special services that can improve outcomes for children with ASD.

**Public Health Action:**

Efforts to increase awareness of ASD and improve the identification of ASD by community providers can facilitate early diagnosis of children with ASD. Heterogeneity of results across sites suggests that community-level differences in evaluation and diagnostic services as well as access to data sources might affect estimates of ASD prevalence and age of identification. Continuing improvements in providing developmental evaluations to children as soon as developmental concerns are identified might result in earlier ASD diagnoses and earlier receipt of services, which might improve developmental outcomes.

## Introduction

Autism spectrum disorder (ASD) is a developmental disability marked by social and communication impairments, as well as restricted interests and repetitive behaviors (*1*). ASD prevalence has been measured by special education and other administrative records (*2*–*4*), national surveys (*5*–*9*), and active public health surveillance conducted through the Metropolitan Atlanta Developmental Disabilities Surveillance Program (MADDSP) and its extended surveillance network, the Autism and Developmental Disabilities Monitoring (ADDM) Network (*10*–*17*). ASD prevalence was first measured by CDC among children aged 3–10 years children by MADDSP in 1996 (*16*). In that analysis, the peak prevalence of ASD was determined to be at age 8 years. Therefore, subsequent to that report, CDC has reported ASD prevalence among children aged 8 years based on data collected every 2 years from 2000 through 2014. Surveillance was conducted by MADDSP and other sites across the United States that participated in the ADDM Network. The most recent ASD prevalence estimate from the ADDM Network was 16.8 per 1,000 children aged 8 years in 2014 (*13*), compared with 14.5 per 1,000 in 2012 (*14*) and 14.7 per 1,000 in 2010 (*15*).

Measuring ASD prevalence and age at diagnosis in elementary school–aged children is expected to yield the most complete information on ASD prevalence and characteristics (*13*–*15*); however, measuring ASD prevalence in preschool-aged children provides more timely assessment of efforts to increase awareness and early detection of ASD. Evidence linking early treatment for ASD with improved outcomes (*18*–*21*) implies that an absence or delay in ASD identification could adversely affect children by delaying interventions and initiation of special services. The American Academy of Pediatrics supports early identification in their recommendation that all children receive ASD screening at ages 18 and 24 months (*22*). Each state has programs to identify children with disabilities and provide special services from birth through age 2 years; children at risk for or with disabilities are eligible for early intervention services through part C of the Individuals with Disabilities Education Act (IDEA) (http://idea.ed.gov). Children aged ≥3 years with disabilities are eligible for evaluation and special education services through part B of IDEA, and these services are provided by public school systems (http://idea.ed.gov).

This report describes ASD prevalence estimates and characteristics among children aged 4 years in the Early ADDM Network for 2010, 2012, and 2014. Selected trend analyses also are presented. The findings in this report can be used by pediatric health care providers, early intervention service providers, therapists, school psychologists, educators, researchers, policymakers, and program administrators seeking to understand and provide for the needs of persons with ASD and their families. These data can be used to help plan for service needs and initiate and implement policies that promote early identification of children with ASD.

## Methods

To estimate the prevalence of ASD in a younger age group, seven of the 13 ADDM sites that conducted ASD surveillance among children aged 8 years during 2010, 2012, 2014 (or all these years) also collected ASD surveillance data for children aged 4 years. These sites are collectively known as the Early ADDM Network. The data for children aged 4 years were collected in subsets of the ADDM geographic areas for children aged 8 years. 

### Study Sites

**FIGURE 1:**
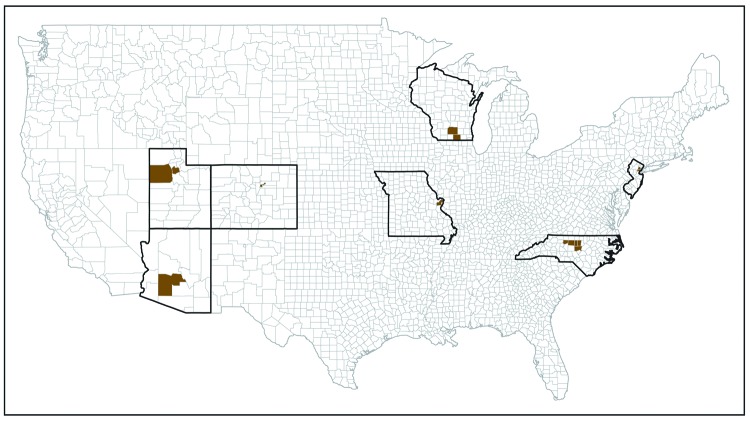
Early Autism and Developmental Disabilities Monitoring Network surveillance areas — seven sites, United States, 2010, 2012, and 2014

The ADDM Network uses a multisite, multiple-source, records-based surveillance method based on a model developed by CDC’s MADDSP (*16*,*23*). In 2010, 2012, and 2014, a total of 13 sites contributed data to the ADDM Network of ASD surveillance among children aged 8 years for at least 1 year (Alabama, Arizona, Arkansas, Colorado, Georgia, Maryland, Minnesota, Missouri, New Jersey, North Carolina, Tennessee, Utah, and Wisconsin). As part of the Early ADDM Network, seven of these sites also conducted ASD surveillance and reported data for children aged 4 years for at least 1 year. The Early ADDM Network included areas of Arizona, Colorado, Missouri, New Jersey, North Carolina, Utah, and Wisconsin (Figure 1). Five Early ADDM sites participated in 2010 and 2012, and six sites participated in 2014. Three Early ADDM sites (Arizona, Missouri, and New Jersey) contributed data and had consistent data sources in all 3 surveillance years.

Because of resource constraints, Early ADDM surveillance was not conducted for the total geographic area covered by each study site’s ADDM surveillance for children aged 8 years; rather, each Early ADDM Network surveillance area was a subset of the site’s total ADDM surveillance area. Each Early ADDM surveillance area included at least 8,000 children aged 4 years and a similar number of children aged 8 years. In comparison, the total ADDM surveillance areas for children aged 8 years for each site included 9,767–51,161 children. The Early ADDM surveillance areas were not random subsets of the total surveillance areas for the respective sites but were selected to form areas of full counties or school districts, within the total ADDM surveillance area that met or exceeded the minimum population size of 8,000 children aged 4 years. Therefore, prevalence estimates for children aged 4 years generated by the Early ADDM Network should not be interpreted as being representative of the prevalence among children aged 4 years for the total ADDM study area at a given site.

Children included in this analysis were born in 2006, 2008, or 2010 for the surveillance years 2010, 2012, and 2014, respectively, and had a parent or guardian who lived in the Early ADDM Network surveillance area during all or part of the specific surveillance year. Participating Early ADDM sites were selected through a competitive review process and were not selected to be nationally representative. A diverse population was preferred during the review process. Each ADDM site functioned as a public health authority under HIPAA (the Health Insurance Portability and Accountability Act of 1996) and met applicable local Institutional Review Board, privacy, and confidentiality requirements (*24*).

### Case Ascertainment

ADDM is an active surveillance system that does not depend on family or professional reporting of an existing ASD diagnosis or classification to determine ASD case status. Case determination is a two-phase process. The first phase involves review and abstraction of records at multiple data sources in the community. In the second phase, all abstracted evaluations are compiled and reviewed by trained study personnel to determine ASD case status. Data sources are categorized as either 1) education source type, including evaluations to determine eligibility for special education services or 2) health care source type, including diagnostic and developmental evaluations. Evaluations must have been performed by a qualified professional, such as a psychologist, physician, physical therapist, occupational therapist, speech or language pathologist, or educator. Children’s records are screened from multiple data sources to determine eligibility for inclusion as a potential case. Developmental assessments completed by a wide range of health care and education providers are reviewed. All Early ADDM Network sites had agreements in place to access records at health care sources. Special education records (for children aged ≥3 years) were reviewed in Arizona, Colorado, New Jersey, North Carolina, and Utah, and early intervention records (for children aged 0 to <3 years) were reviewed in New Jersey, North Carolina, Utah, and Wisconsin; in Wisconsin, early intervention records were reviewed for 2014 only. The ADDM Network review only includes existing records, not clinical examinations of children.

In the first phase of surveillance, ADDM Network sites identify source records to review according to a child’s year of birth and either 1) eligibility classifications in special education or early intervention, or 2) *International Classification of Diseases, Ninth Revision, Clinical Modification* (ICD-9-CM) or *International Classification of Diseases, Tenth Revision* (ICD-10) billing codes for select childhood disabilities or conditions. Children’s records are screened to confirm year of birth and residency in the surveillance area at some time during the surveillance year. For children meeting age and residency requirements, the source files are screened for certain behavioral or diagnostic descriptions defined by ADDM as triggers for abstraction (e.g., child does not initiate interactions with others, prefers to play alone or engage in solitary activities, or has received a documented ASD diagnosis). If abstraction triggers are found, evaluation information from birth through the current surveillance year is abstracted into a single composite record for each child. The composite record includes comprehensive evaluations by qualified professionals from birth through the end of the year when the child reaches either age 4 or 8 years.

In the second phase of surveillance, the abstracted comprehensive evaluations are deidentified and reviewed systematically by clinicians who have undergone standardized training to determine ASD case status using a coding scheme based on the *Diagnostic and Statistical Manual of Mental Disorders, 4th Edition, Text Revision* (DSM-IV-TR) (*25*) criteria for ASD. These clinicians review each comprehensive evaluation and code the behavioral descriptors according to the DSM-IV-TR criteria represented by the descriptor. 

### Surveillance Case Definition

Children included in this analysis were born in 2006, 2008, or 2010 for the surveillance years 2010, 2012, and 2014, respectively, and had a parent or guardian who lived in the Early ADDM Network surveillance area during all or part of the specific surveillance year. A child aged 4 or 8 years met the surveillance case definition for ASD if behaviors described within one or more comprehensive evaluations were consistent with the DSM-IV-TR diagnostic criteria for any of the following conditions: autistic disorder, pervasive developmental disorder–not otherwise specified (PDD-NOS, including atypical autism), or Asperger disorder (Box 1). An ASD diagnosis alone was not sufficient to meet the DSM-IV-TR surveillance case definition but was considered during the clinician review process, along with behavioral criteria. Most records were reviewed by a single person, although clinicians were able to request a second review if they were uncertain about whether the behaviors were consistent with the DSM-IV-TR diagnostic criteria. Children could have been disqualified from meeting the case definition if their behaviors met the surveillance case definition but one or more clinician reviewers judged that sufficient information existed to rule out ASD, information to support an ASD diagnosis was conflicting or insufficient, or that one or more other diagnosed conditions better accounted for their symptoms.

BOX 1 **Surveillance case definition based on behavioral criteria for diagnosis of autism spectrum disorder: *Diagnostic and Statistical Manual of Mental Disorders,* *4th Edition, Text Revision***

**Table:** 

**DSM-IV-TR behavioral criteria**	
Social	1a. Marked impairment in the use of multiple nonverbal behaviors, such as eye-to-eye gaze, facial expression, body postures, and gestures to regulate social interaction 1b. Failure to develop peer relationships appropriate to developmental level 1c. A lack of spontaneous seeking to share enjoyment, interests, or achievements with other people (e.g., by a lack of showing, bringing, or pointing out objects of interest) 1d. Lack of social or emotional reciprocity
Communication	2a. Delay in, or total lack of, the development of spoken language (not accompanied by an attempt to compensate through alternative modes of communication, such as gesture or mime) 2b. In individuals with adequate speech, marked impairment in the ability to initiate or sustain a conversation with others 2c. Stereotyped and repetitive use of language or idiosyncratic language 2d. Lack of varied, spontaneous make-believe play or social imitative play appropriate to developmental level
Restricted behavior/Interest	3a. Encompassing preoccupation with one or more stereotyped and restricted patterns of interest that is abnormal either in intensity or focus 3b. Apparently inflexible adherence to specific, nonfunctional routines, or rituals 3c. Stereotyped and repetitive motor mannerisms (e.g., hand or finger flapping or twisting, or complex whole body movements) 3d. Persistent preoccupation with parts of objects
Developmental history	Child had identified delays or any concern with development in the following areas at or before the age of 3 years: Social, Communication, Behavior, Play, Motor, Attention, Adaptive, or Cognitive
Autism discriminators	Oblivious to children Oblivious to adults or others Rarely responds to familiar social approach Language primarily echolalia or jargon Regression/loss of social, language, or play skills Previous ASD diagnosis, whether based on DSM-IV-TR or DSM-5 diagnostic criteria Lack of showing, bringing, etc. Little or no interest in others Uses others as tools Repeats extensive dialog Absent or impaired imaginative play Markedly restricted interests Unusual preoccupation Insists on sameness Nonfunctional routines Excessive focus on parts Visual inspection Movement preoccupation Sensory preoccupation
**DSM-IV-TR surveillance case definition**	
At least six behaviors coded with a minimum of two Social, one Communication, and one Restricted Behavior/Interest; AND evidence of developmental delay or concern at or before the age of 3 years OR At least two behaviors coded with a minimum of one Social and either one Communication and/or one Restricted Behavior/Interest; AND at least one autism discriminator coded **Note:** A child might be disqualified from meeting the DSM-IV-TR surveillance case definition for ASD if, based on the clinical judgment of one or more reviewers, there is insufficient or conflicting information in support of ASD, sufficient information to rule out ASD, or if one or more other diagnosed conditions better account for the child’s symptoms.	

**Abbreviations**: ASD = autism spectrum disorder; DSM-IV-TR = *Diagnostic and Statistical Manual of Mental Disorders, Fourth Edition, Text Revision;* DSM-5= *Diagnostic and Statistical Manual of Mental Disorders, Fifth Edition.*

Updated behavioral criteria for an ASD diagnosis were published in 2013 in the *Diagnostic and Statistical Manual of Mental Disorders, 5th Edition* (DSM-5) (*1*). To determine the effect of the updated DSM-5 behavioral criteria on ASD prevalence, a revised surveillance case definition (Box 2) also was used to classify cases for the 2014 surveillance year. A child aged 4 or 8 years met the DSM-5 surveillance case definition if behaviors described within one or more comprehensive evaluations were consistent with the DSM-5 diagnostic criteria or if an ASD diagnosis had been documented, regardless of whether the behavioral criteria had been met. Most records were reviewed by a single person, although clinicians were able to request a second review if they were uncertain about whether the behaviors were consistent with the DSM-5 diagnostic criteria. Children could have been disqualified from meeting the case definition if their behaviors met the surveillance case definition but one or more clinician reviewers judged that sufficient information existed to rule out ASD, information to support an ASD diagnosis was conflicting or insufficient, or that one or more other diagnosed conditions better accounted for their symptoms.

BOX 2 Surveillance case definition based on behavioral criteria for diagnosis of autism spectrum disorder*: *Diagnostic and Statistical Manual of Mental Disorders, 5th Edition*

**Table:** 

**DSM-5 behavioral criteria**	
A. Persistent deficits in social communication and social interaction	A1: Deficits in social emotional reciprocity A2. Deficits in nonverbal communicative behaviors A3. Deficits in developing, maintaining, and understanding relationships
B. Restricted, repetitive patterns of behavior, interests, or activities, currently or by history	B1: Stereotyped or repetitive motor movements, use of objects or speech B2. Insistence on sameness, inflexible adherence to routines, or ritualized patterns of verbal or nonverbal behavior B3. Highly restricted interests that are abnormal in intensity or focus B4. Hyperreactivity or hyporeactivity to sensory input or unusual interest in sensory aspects of the environment
Historical pervasive developmental disorder diagnosis	Any ASD diagnosis documented in a comprehensive evaluation, including a DSM-IV diagnosis of autistic disorder, Asperger disorder, or pervasive developmental disorder–not otherwise specified
**DSM-5 surveillance case definition**	
All three behavioral criteria coded under part A, and at least two behavioral criteria coded under part B OR Any ASD diagnosis documented in a comprehensive evaluation, whether based on DSM-IV-TR or DSM-5 diagnostic criteria **Note:** A child might be disqualified from meeting the DSM-5 surveillance case definition for ASD if, based on the clinical judgment of one or more reviewers, there is insufficient or conflicting information in support of ASD, sufficient information to rule out ASD, or if one or more other diagnosed conditions better account for the child’s symptoms.	

**Abbreviations**: ASD = autism spectrum disorder; DSM-IV = *Diagnostic and Statistical Manual of Mental Disorders, Fourth Edition;* DSM-IV-TR = *Diagnostic and Statistical Manual of Mental Disorders, Fourth Edition, Text Revision;* DSM-V = *Diagnostic and Statistical Manual of Mental Disorders, Fifth Edition.*

*DSM-5 also includes a previous DSM-IV diagnosis of ASD as a sole criterion for a clinical diagnosis.

In this report, most results are based on the DSM-IV-TR surveillance case definition for consistency and comparison across surveillance years. Results comparing ASD prevalence using both DSM-IV-TR and DSM-5 surveillance case definitions are included for 2014.

### Descriptive Characteristics

Demographic information, including sex and race/ethnicity, was abstracted. Data on sex were available for all children. Data on race/ethnicity were missing for <5% of children across all years, age groups, and surveillance sites. Children with missing race/ethnicity data were not included in analyses stratified by race/ethnicity but were included in analyses of all children combined. Each site obtained vital records data for the relevant birth year, which were linked to surveillance data to obtain supplemental information on race/ethnicity and other demographic characteristics.

Diagnostic summaries from each evaluation were abstracted for each child, including notation of any ASD diagnosis by subtype. Children were considered to have an ASD diagnosis from a community provider if they received a diagnosis of autistic disorder, Asperger disorder, PDD-NOS, or ASD that was documented in an abstracted evaluation at any time from birth through the year when they reached age 4 or 8 years. The age at each documented ASD diagnosis from a community provider was abstracted, as well as the age at each comprehensive developmental evaluation. These data were used to determine the age at the earliest known ASD diagnosis, if any, and the age at the first comprehensive developmental evaluation. Data on age at first evaluation were restricted to children who were born in the state where the ADDM Network site was located to avoid bias from the inability to locate early evaluations for children who moved into the study area. In-state birth was determined through a successful match to a birth certificate from that state. If no birth certificate was found, the child was presumed to have been born outside the state where the surveillance site was located. Because all children had at least one evaluation, the age at the first evaluation was available for all children and is reported as the median age (in months), along with the percentage of children with a first evaluation by age 36 months. This age was chosen to align with the Healthy People 2020 (http://www.healthypeople.gov/2020/default.aspx) goal of increasing the percentage of children with ASD who receive their first developmental evaluation by the age of 36 months. Not all children had a documented ASD diagnosis from a community provider; a total of 272 (34.7%), 318 (35.1%), and 508 (42.1%) children had no ASD diagnosis for 2010, 2012, and 2014, respectively. The age at earliest known ASD diagnosis could be described only for those children with a documented diagnosis and is reported as the median age in months. Ages of <6 months at earliest known ASD diagnosis were excluded for implausibility (n = 2).

Data were collected on results of standardized tests of intellectual ability found in children’s records, and children were considered to have an intellectual disability if they had a score of ≤70 on their most recent test. Data on intellectual ability were included for sites for which ≥60% of children meeting the ASD surveillance case definition had an intellectual ability test score. Among those sites, children without a test score were categorized as having unknown intellectual ability (n = 114 [18.8%], n = 114 [21.5%], and n = 225 [25.7%] for 2010, 2012, and 2014, respectively). Uncertainty surrounding the reliability of measurement of intellectual ability in early childhood prevents further subclassification of intellectual ability (*26*,*27*).

### Quality Assurance

All Early ADDM sites follow the same quality assurance conventions established by the ADDM Network. For the first phase of ADDM, screening and abstraction of source records are checked periodically for accuracy. For the second phase, interrater reliability receives ongoing monitoring, with a blinded, random 10% sample of abstracted records that are scored independently by two reviewers. Across surveillance years, the final average interrater agreements for determining ASD surveillance case status in the Early ADDM study sites ranged from 87.3% (κ = 0.74) to 91.1% (κ = 0.81) among children aged 4 years and from 89.2% (κ = 0.77) to 91.0% (κ = 0.80) among those aged 8 years.

### Analytic Methods

The objectives of this report are to describe ASD prevalence and characteristics among children aged 4 years in the Early ADDM Network for 2010, 2012, and 2014, including 1) overall prevalence and prevalence by sex and race/ethnicity; 2) characteristics of children aged 4 years with ASD, including age at first developmental evaluation, age at ASD diagnosis, and cognitive function; and 3) trends in ASD prevalence and characteristics in the three Early ADDM sites with data and consistent data sources for all 3 surveillance years (2010, 2012, and 2014), including comparisons with children aged 8 years living in the same geographic areas. Data for 2010 were previously published (*28*) but are included in the results to provide a comprehensive representation of ASD prevalence and characteristics for all the years of Early ADDM Network surveillance, as well as a comparison among children from the sites with data from all 3 surveillance years.

The prevalence estimate of ASD among children aged 4 years was calculated as the number of children aged 4 years who met the ASD surveillance case definition in the Early ADDM Network sites in 2010, 2012, and 2014 divided by the number of children aged 4 years living in the surveillance areas according to the 2010 decennial bridged-race population estimates (*29*), the vintage 2014 postcensal bridged-race population estimates for 2012 (http://www.cdc.gov/nchs), and the vintage 2016 postcensal bridged-race population estimates for 2014 (http://www.cdc.gov/nchs). In Arizona and Utah, the surveillance area included some but not all of the school districts in two counties (Maricopa and Salt Lake counties, respectively). Therefore, investigators developed a method using census and school district data to estimate the numbers of children aged 4 and 8 years living in these surveillance areas. Detailed methods are provided (Appendix). Overall prevalence estimates included all children identified with ASD regardless of sex, race/ethnicity, or intellectual ability and therefore were unaffected by the availability of these data elements.

Statistical tests and 95% confidence interval (CI) estimates were derived under the assumption that the observed counts of ASD surveillance cases were sampled from an underlying Poisson distribution. Because previous ADDM Network reports presented CIs based on an underlying Poisson distribution with an asymptotic approximation to the normal, slight differences might exist between those and the exact Poisson confidence intervals presented in this report. Generalized linear models with a Poisson distribution were used to calculate prevalence ratios (PRs) and CIs. Pearson chi-square tests were used to examine frequency differences in the characteristics of children with ASD by surveillance area, sex, race/ethnicity, and intellectual ability; ASD prevalence was estimated both for children aged 4 years and 8 years living in the Early ADDM surveillance areas. Because the data for children aged 8 years are restricted to this smaller area, the estimates for those aged 8 years do not match those previously published from the ADDM Network reports on ASD prevalence and characteristics (*13*–*15*). Trend analyses for ASD prevalence were restricted to the three sites (Arizona, Missouri, and New Jersey) with data and consistent data sources for all 3 years; trends in the proportion of children with ASD who had co-occurring intellectual disabilities were restricted to the two sites with data for all 3 years (Arizona and New Jersey). Cochran-Armitage trend tests were used to estimate the significance of changes in ASD characteristics over the 2010–2014 period. The nonparametric median test was used to determine differences in median age at first developmental evaluation and earliest known ASD diagnosis from 2010 to 2014 and by sex and race/ethnicity within surveillance years. PRs with CIs that did not include 1.00 were used to assess whether ASD prevalence was higher in one population than another. For results from chi-square, Cochran-Armitage, and median tests, a p value of <0.05 was considered significant. Analyses were performed using SAS (version 9.4; SAS Institute).

## Results

### Population Distribution

The overall Early ADDM Network geographic surveillance area includes the seven sites that participated in at least one surveillance year (Figure 1). The Early ADDM Network comprised a population from 58,467 (2010) to 70,887 (2014) children aged 4 years and 56,727 (2010) to 71,928 (2014) children aged 8 years (Supplemental Table 1, https://stacks.cdc.gov/view/cdc/76016). The distribution of children by race/ethnicity varied across the sites. Among children aged 4 years, the percentage of white children ranged from 29.4% (New Jersey in 2014) to 70.9% (Wisconsin in 2014), and the percentage of black children ranged from 3.5% (Arizona in 2012 and 2014) to 33.1% (New Jersey in 2014). The percentage of Hispanic children ranged from 4.5% (Missouri in 2014) to 47.3% (Colorado in 2014). American Indian/Alaska Native children comprised 0.2%–3.1% of the total population, and Asian/Pacific Islander children comprised 2.7%–6.5%. The population distribution by race/ethnicity across sites was similar for children aged 8 years. Aggregating data across sites for each surveillance year, the total percentages by race/ethnicity among children aged 4 years ranged from 46.8% to 51.9% for white (in 2014 and 2010, respectively), 19.1% to 22.7% for black (in 2010 and 2014, respectively), 23.2% to 25.1% for Hispanic (in 2010 and 2014, respectively), 4.7% to 5.0% for Asian/Pacific Islander (in 2014 and 2012, respectively), and 0.7% to 0.9% for American Indian/Alaska Native (in 2014 and 2010–2012, respectively), with similar percentages among children aged 8 years.

### Overall ASD Prevalence Among Children Aged 4 Years

Aggregating data across participating surveillance sites for each year, the estimated prevalence of ASD among children aged 4 years was 13.4 per 1,000 in 2010, 15.3 in 2012, and 17.0 in 2014 (Table 1). Prevalence ranged from 8.1 per 1,000 children aged 4 years in Missouri (2012) to 28.4 in New Jersey (2014). For each year, aggregated ASD prevalence was higher for study sites that reviewed education and health care records rather than health care records alone (Table 1); PRs for sites that reviewed both types compared with only health care records were 1.8 (95% CI: 1.6–2.2) in 2010, 1.6 (95% CI: 1.4–1.8) in 2012, and 1.7 (95% CI: 1.5–2.0) in 2014 (data not shown).

**TABLE 1:** Prevalence* of autism spectrum disorder among children aged 4 years — Autism and Developmental Disabilities Monitoring Network, seven sites, United States, 2010, 2012, and 2014

Year, record source, and site	No. with ASD	Total population	Prevalence (95% CI)
**2010**			
Health care and education			
Arizona^†^	123	9,265	13.3 (11.0–15.8)
New Jersey**^§,^**^¶^	352	17,860	19.7 (17.7–21.9)
Utah^¶,^**	132	10,944	12.1 (10.1–14.3)
**Total**	**607**	**38,069**	**15.9 (14.7–17.3)**
Health care only			
Missouri^††^	103	12,095	8.5 (7.0–10.3)
Wisconsin**^§§^**	73	8,303	8.8 (6.9–11.1)
**Total**	**176**	**20,398**	**8.6 (7.4–10.0)**
**Combined total**	**783**	**58,467**	**13.4 (12.5–14.4)**
**2012**			
Health care and education			
Arizona	128	9,621	13.3 (11.1–15.8)
New Jersey^¶^	403	18,223	22.1 (20.0–24.4)
Utah^¶^	152	11,398	13.3 (11.3–15.6)
**Total**	**683**	**39,242**	**17.4 (16.1–18.8)**
Health care only			
Missouri	96	11,878	8.1 (6.5–9.9)
Wisconsin	128	8,336	15.4 (12.8–18.3)
**Total**	**224**	**20,214**	**11.1 (9.7–12.6)**
**Combined total**	**907**	**59,456**	**15.3 (14.3–16.3)**
**2014**			
Health care and education			
Arizona	130	9,624	13.5 (11.3–16.0)
Colorado^¶¶^	113	8,438	13.4 (11.0–16.1)
New Jersey^¶^	514	18,112	28.4 (26.0–30.9)
North Carolina^¶,^***	231	14,893	15.5 (13.6–17.6)
**Total**	**988**	**51,067**	**19.3 (18.2–20.6)**
Health care only			
Missouri	112	11,613	9.6 (7.9–11.6)
Wisconsin^¶^	108	8,207	13.2 (10.8–15.9)
**Total**	**220**	**19,820**	**11.1 (9.7–12.7)**
**Combined total**	**1,208**	**70,887**	**17.0 (16.1–18.0)**

**Abbreviations:** ASD = autism spectrum disorder; CI = confidence interval.

* Prevalence per 1,000 children aged 4 years living in the surveillance areas according to the 2010 decennial bridged-race population estimates (US Census Bureau. Census summary file 1: Tables PCT12H–PCT12O. Washington, DC: US Census Bureau; 2010), the vintage 2014 postcensal bridged-race population estimates for 2012 (http://www.cdc.gov/nchs), and the vintage 2016 postcensal bridged-race population estimates for 2014 (http://www.cdc.gov/nchs).

^†^ Part of one county in metropolitan Phoenix for 2010, 2012, and 2014.

**^§^** Essex and Union counties for 2010, 2012, and 2014.

^¶^ Site also reviewed records from early intervention sources.

** Tooele County, part of Salt Lake County, for 2010 and 2012 only.

^††^ One county in metropolitan St. Louis for 2010, 2012, and 2014.

^§§^ Dane and Rock counties for 2010, 2012, and 2014.

^¶¶^ One county in metropolitan Denver for 2014 only.

*** Alamance, Chatham, Guilford, Orange, and Forsyth counties for 2014 only.

### ASD Prevalence Among Children Aged 4 Years by Sex and Race/Ethnicity

Across all sites and years, ASD prevalence per 1,000 boys aged 4 years ranged from 12.2 in Missouri (2010) to 44.0 in New Jersey (2014) (Table 2). Prevalence per 1,000 girls aged 4 years ranged from 3.2 in Missouri (2012) to 12.1 in New Jersey (2014). Male-to-female PRs indicated ASD prevalence was higher among boys than girls in all sites and years, ranging from 2.6 (Arizona and Wisconsin in 2010) to 5.2 boys per one girl (Colorado in 2014).

**TABLE 2:** Prevalence* of autism spectrum disorder among children aged 4 years, by sex — Early Autism and Developmental Disabilities Monitoring Network, seven sites, United States, 2010, 2012, and 2014

Year, record source, and site	Sex		Prevalence ratio, male to female (95% CI)^†^
	Male	Female	
	Prevalence (95% CI)	Prevalence (95% CI)	
**2010**			
Health care and education			
Arizona	18.9 (15.2–23.3)	7.3 (5.0–10.3)	2.6 (1.7–3.9)
New Jersey**^§^**	31.7 (28.1–35.5)	7.2 (5.5–9.2)	4.4 (3.3–5.8)
Utah**^§^**	17.9 (14.6–21.7)	5.9 (4.0–8.3)	3.1 (2.0–4.6)
Health care only			
Missouri	12.2 (9.6–15.3)	4.6 (3.0–6.7)	2.7 (1.7–4.1)
Wisconsin	12.5 (9.4–16.4)	4.8 (2.9–7.4)	2.6 (1.6–4.4)
**2012**			
Health care and education			
Arizona	21.3 (17.5–25.8)	4.6 (2.8–7.0)	4.7 (2.9–7.5)
New Jersey**^§^**	33.6 (30.0–37.5)	9.9 (8.0–12.2)	3.4 (2.7–4.3)
Utah**^§^**	20.7 (17.2–24.8)	5.7 (3.9–8.1)	3.6 (2.5–5.4)
Health care only			
Missouri	12.9 (10.2–16.2)	3.2 (1.9–5.0)	4.0 (2.4–6.7)
Wisconsin	23.7 (19.4–28.8)	6.4 (4.2–9.4)	3.7 (2.4–5.7)
**2014**			
Health care and education			
Arizona	21.3 (17.4–25.8)	5.2 (3.3–7.7)	4.1 (2.7–6.4)
Colorado	22.3 (18.1–27.3)	4.3 (2.5–6.8)	5.2 (3.1–8.6)
New Jersey**^§^**	44.0 (39.9–48.5)	12.1 (10.0–14.7)	3.6 (2.9–4.5)
North Carolina**^§^**	24.7 (21.3–28.5)	5.8 (4.2–7.8)	4.2 (3.0–5.9)
Health care only			
Missouri	14.2 (11.3–17.5)	4.8 (3.2–7.0)	3.0 (1.9–4.6)
Wisconsin**^§^**	20.8 (16.7–25.6)	4.8 (2.9–7.6)	4.3 (2.6–7.1)

**Abbreviations:** CI = confidence interval; PR = prevalence ratio.

* Prevalence per 1,000 children age 4 years living in the surveillance areas according to the 2010 decennial bridged-race population estimates (US Census Bureau. Census summary file 1: Tables PCT12H–PCT12O. Washington, DC: US Census Bureau; 2010), the vintage 2014 postcensal bridged-race population estimates for 2012 (http://www.cdc.gov/nchs), and the vintage 2016 postcensal bridged-race population estimates for 2014 (http://www.cdc.gov/nchs).

^†^ Results for PRs considered statistically significant when the CI excludes the null value (PR = 1.0).

^§^ Site also reviewed records from early intervention sources.

Across all study sites and years for children aged 4 years, prevalence among white children ranged from 7.7 per 1,000 in Missouri (2014) to 29.3 in New Jersey (2014) (Table 3). Prevalence among black children ranged from 3.8 per 1,000 in Missouri (2010) to 24.7 in New Jersey (2014). Prevalence among Hispanic children ranged from 9.1 per 1,000 (in Arizona (2010) to 28.2 in New Jersey (2014). In 2010, white children had a higher ASD prevalence than Hispanic children in Arizona (PR = 1.7) and black children in Missouri (PR = 2.5); no other differences were observed by race/ethnicity.

**TABLE 3:** Prevalence* of autism spectrum disorder among children aged 4 years, by race/ethnicity — Early Autism and Developmental Disabilities Monitoring Network, seven sites, United States, 2010, 2012, and 2014

Year, record source, and site	Prevalence (95% CI)	Prevalence (95% CI)	Prevalence (95% CI)	Prevalence ratio (95% CI)^†^	
	White, non-Hispanic	Black, non-Hispanic	Hispanic	White to black	White to Hispanic
**2010 **					
Health care and education					
Arizona	15.7 (12.4–19.7)	—^¶^	9.1 (6.2–12.9)	—	1.7 (1.1–2.6)
New Jersey^§^	18.9 (15.5–22.7)	16.7 (13.6–20.4)	22.5 (18.6–27.0)	1.1 (0.9–1.5)	0.8 (0.6–1.1)
Utah^§^	14.0 (11.3–17.2)	—	9.8 (6.7–13.8)	—	1.4 (1.0–2.1)
Health care only					
Missouri	9.3 (7.2–11.9)	3.8 (2.1–6.4)	14.4 (6.2–28.4)	2.5 (1.4–4.4)	0.6 (0.3–1.4)
Wisconsin	8.2 (6.0–10.9)	—	—	—	—
**2012**					
Health care and education					
Arizona	14.5 (11.4–18.1)	20.7 (8.3–42.7)	9.9 (6.8–13.8)	0.7 (0.3–1.5)	1.5 (1.0–2.2)
New Jersey^§^	24.2 (20.3–28.5)	19.3 (15.9–23.1)	22.3 (18.6–26.6)	1.3 (1.0–1.6)	1.1 (0.8–1.4)
Utah^§^	14.3 (11.5–17.5)	—	11.3 (8.1–15.4)	—	1.3 (0.9–1.8)
Health care only					
Missouri	8.3 (6.3–10.8)	7.6 (5.1–11.0)	—	1.1 (0.7–1.7)	—
Wisconsin	13.9 (11.0–17.2)	7.6 (3.0–15.6)	15.6 (9.1–24.9)	1.8 (0.8–4.0)	0.9 (0.5–1.5)
**2014**					
Health care and education					
Arizona	15.2 (12.0–18.8)	14.9 (4.8–34.8)	11.1 (7.8–15.4)	1.0 (0.4–2.5)	1.4 (0.9–2.0)
Colorado	11.7 (8.3–16.2)	18.0 (10.5–28.9)	12.3 (9.1–16.2)	0.7 (0.4–1.2)	1.0 (0.6–1.5)
New Jersey^§^	29.3 (24.8–34.2)	24.7 (20.9–29.0)	28.2 (24.1–32.8)	1.2 (0.9–1.5)	1.0 (0.8–1.3)
North Carolina^§^	14.6 (11.8–17.8)	16.8 (13.2–21.0)	10.9 (7.5–15.3)	0.9 (0.6–1.2)	1.3 (0.9–2.0)
Health care only					
Missouri	7.7 (5.8–10.1)	10.4 (7.3–14.3)	—	0.7 (0.5–1.1)	—
Wisconsin^§^	13.1 (10.3–16.3)	9.7 (4.2–19.1)	11.5 (5.9–20.0)	1.3 (0.6–2.8)	1.1 (0.6–2.1)

**Abbreviations:** CI = confidence interval; PR = prevalence ratio.

* Prevalence per 1,000 children aged 4 years living in the surveillance areas according to the 2010 decennial bridged-race population estimates (US Census Bureau. Census summary file 1: Tables PCT12H–PCT12O. Washington, DC: US Census Bureau; 2010), the vintage 2014 postcensal bridged-race population estimates for 2012 (http://www.cdc.gov/nchs), and the vintage 2016 postcensal bridged-race population estimates for 2014 (http://www.cdc.gov/nchs).

^†^ Results for PRs considered statistically significant when the CI excludes the null value (PR = 1.0).

^§^ Site also reviewed records from early intervention sources.

^¶^ Estimates suppressed due to small cell sizes (N<5).

### Frequency of Co-Occurring Intellectual Disabilities Among Children Aged 4 and 8 Years

Scores on intellectual ability tests were available for at least 60% of children in four sites for at least one surveillance year (Arizona, New Jersey, North Carolina, and Utah). These sites all reviewed education and health care records. In the two sites (Arizona and New Jersey) with data for all surveillance years, the percentage of children aged 4 years with ASD who had co-occurring intellectual disabilities was stable over time at 47.0%, 43.6%, and 46.0% in 2010, 2012, and 2014, respectively (test for trend p value = 0.84) and also was stable over time among both boys and girls (Table 4). The proportion of children with ASD who had co-occurring intellectual disabilities was significantly higher among children aged 4 years than among those aged 8 years across all sites and surveillance years, with the exception of Arizona (2010) (Supplemental Table 2, https://stacks.cdc.gov/view/cdc/76016).

**TABLE 4:** Number and percentage of children with co-occurring intellectual disability* among children aged 4 years with autism spectrum disorder, by site, sex, and year — Early Autism and Developmental Disabilities Monitoring Network, four sites,^†^ United States, 2010, 2012, and 2014

Site and sex	2010		2012		2014		2010–2014
	Children with cognitive test scores	Children with co-occurring intellectual disability	Children with cognitive test scores	Children with co-occurring intellectual disability	Children with cognitive test scores	Children with co-occurring intellectual disability	p value^§^
	No. (% of children with ASD)	No. (%)	No. (% of children with ASD)	No. (%)	No. (% of children with ASD)	No. (%)	
**Site**							
Arizona	105 (85.4)	43 (41.0)	80 (62.5)	33 (41.3)	90 (69.2)	45 (50.0)	0.21
New Jersey	291 (82.7)	143 (49.1)	337 (83.6)	149 (44.2)	418 (81.3)	189 (45.2)	0.34
North Carolina	—^¶^	—	—	—	142 (61.5)	64 (45.1)	—
Utah	97 (73.5)	40 (41.2)	—	—	—	—	—
Sex**							
Male	312 (82.3)	152 (48.7)	334 (79.1)	146 (43.7)	409 (79.9)	191 (46.7)	0.65
Female	84 (87.5)	34 (40.5)	83 (76.1)	36 (43.4)	99 (75.0)	43 (43.4)	0.69
Total**	**396 (83.4)**	**186 (47.0)**	**417 (78.5)**	**182 (43.6)**	**508 (78.9)**	**234 (46.1)**	**0.84**

**Abbreviation:** ASD = autism spectrum disorder.

* Defined as a score of ≤70 on the most recent standardized cognitive ability test.

^†^ Including sites for which at least 60% of children with ASD had cognitive ability test score data for at least 1 surveillance year.

^§^ Cochran-Armitage trend test for percentage with intellectual disability; p<0.05 indicates statistical significance.

^¶^ No or insufficient data for site and surveillance year.

** Data restricted to sites with information for all 3 years (Arizona and New Jersey).

### Age at First Comprehensive Developmental Evaluation Among Children Aged 4 Years

Across all participating sites and surveillance years and among children born in the state where the ADDM Network site was located, the percentage of children who received their first comprehensive developmental evaluation by age 36 months ranged from 48.8% (Missouri in 2012) to 88.9% (Wisconsin in 2014) (Table 5). Among the three sites with data and consistent data sources for all 3 years, patterns in the age at the first developmental evaluation varied by site. No trend was observed in Arizona or Missouri. In New Jersey, from 2010 to 2014, the percentage of children who received a first evaluation by age 36 months decreased significantly (from 76.5% to 66.7%). In Wisconsin, the percentage of children who received a first developmental evaluation by age 36 months was higher in 2014 (88.9%), when early intervention records were reviewed, than in 2010 and 2012 (69.0% and 73.4%, respectively). Percentages stratified by sex and race/ethnicity by site are provided (Supplemental Table 3, https://stacks.cdc.gov/view/cdc/76016).

**TABLE 5:** Median age at earliest known comprehensive evaluation and percentage of children evaluated by age 36 months among children aged 4 years with autism spectrum disorder — Early Autism and Developmental Disabilities Monitoring Network, seven sites, United States, 2010, 2012, and 2014

Site and record source	2010			2012			2014			p value*
	Median age (months)	Total no. with ASD	No. (%) with evaluation by 36 months	Median age (months)	Total no. with ASD	No. (%) with evaluation by 36 months	Median age (months)	Total no. with ASD	No. (%) with evaluation by 36 months	
**Health care and education**										
Arizona	34.0	95	58 (61.1)	32.0	110	74 (67.3)	32.5	110	76 (69.1)	0.23
Colorado	—^†^	—^†^	—^†^	—^†^	—^†^	—^†^	34.0	93	75 (80.6)	^—§^
New Jersey	26.0	307	235 (76.5)	29.0	344	271 (78.8)	34.0	403	269 (66.7)	0.002
North Carolina	—^†^	—^†^	—^†^	—^†^	—^†^	—^†^	23.0	198	164 (82.8)	^—§^
Utah	32.0	107	75 (70.1)	32.0	115	72 (62.6)	—^†^	—^†^	—^†^	^—§^
**Health care only**										
Missouri	30.0	88	61 (69.3)	37.0	80	39 (48.8)	29.0	90	67 (74.4)	0.46
Wisconsin	27.5	58	40 (69.0)	29.0	109	80 (73.4)	24.0	90	80 (88.9)	—^¶^

**Abbreviation:** ASD = autism spectrum disorder.

* Cochran-Armitage trend test for proportion with evaluation by age 36 months; p<0.05 indicates statistical significance.

^†^ No data for site and surveillance year.

^§^ Trend not estimated for sites with <3 years of data.

^¶^ Trend not estimated because records were included from early intervention sources for 2014 but not earlier years.

### ASD Diagnosis from a Community Provider Among Children Aged 4 Years

The percentage of children with a documented ASD diagnosis from a community provider ranged from 43.0% in Arizona (2012) to 86.5% in Missouri (2012) but did not vary by sex (Table 6). The median age at first known ASD diagnosis ranged from 28 months in North Carolina (2014) to 39.0 months in Missouri and Wisconsin (2012). Among the three sites with data for all 3 surveillance years and consistent data sources, no significant trends were found in the proportion of children with an ASD diagnosis, overall or by sex

**TABLE 6:** Number and percentage of children aged 4 years with a previous autism spectrum disorder diagnosis and median age at earliest known diagnosis — Early Autism and Developmental Disabilities Monitoring Network, seven sites, United States, 2010, 2012, and 2014

Site and record source	2010			2012			2014			2010–2014
	Total no. with ASD	No. (%) with any ASD diagnosis	Median age (months) of earliest known ASD diagnosis	Total no. with ASD	No. (%) with any ASD diagnosis	Median age (months) of earliest known ASD diagnosis	Total no. with ASD	No. (%) with any ASD diagnosis	Median age (months) of earliest known ASD diagnosis	p value*
**Health care and education**										
Arizona	123	53 (43.1)	35.0	128	55 (43.0)	36.0	130	56 (43.1)	36.0	1.0
Colorado	—^†^	—^†^	—^†^	—^†^	—^†^	—^†^	113	72 (63.7)	31.0	—^§^
New Jersey	352	207 (58.8)	32.5	403	236 (58.6)	35.0	514	292 (56.8)	33.5	0.54
North Carolina	—^†^	—^†^	—^†^	—^†^	—^†^	—^†^	231	107 (46.3)	28.0	—^§^
Utah	132	106 (80.3)	35.0	152	122 (80.3)	35.0	—^†^	—^†^	—^†^	—^§^
**Health care only**										
Missouri	103	84 (81.6)	34.0	96	83 (86.5)	39.0	112	96 (85.7)	36.0	0.41
Wisconsin	73	61 (83.6)	34.0	128	93 (72.7)	39.0	108	77 (71.3)	33.0	—^¶^

**Abbreviation:** ASD = autism spectrum disorder.

*Cochran-Armitage trend test for percentage with any ASD diagnosis; p<0.05 indicates statistical significance.

^†^ No data for site for surveillance year.

^§^ Trend not estimated for sites with <3 years of data.

^¶^ Trend not estimated because records were included from early intervention sources for 2014 but not earlier years.

### Trends in ASD Prevalence Among Children Aged 4 and 8 Years

**FIGURE 2:**
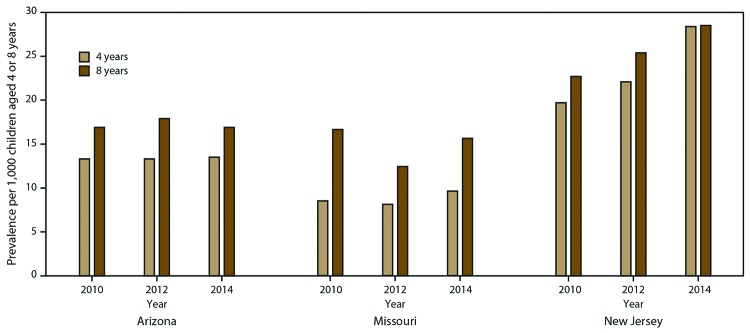
Trends in autism spectrum disorder prevalence* among children aged 4 years and 8 years — Early Autism and Developmental Disabilities Monitoring Network, three sites, United States, 2010, 2012, and 2014 * In Arizona in 2012, the prevalence among children aged 4 years and children aged 8 years was significantly different (p<0.05 for chi-square test). In Missouri, the prevalence was significantly different in all 3 years. (In New Jersey, no differences were significant in any years.)

Four Early ADDM Network sites (Arizona, Missouri, New Jersey, and Wisconsin) participated in all 3 surveillance years; however, Wisconsin reviewed early intervention records in 2014 but not earlier years, whereas data sources for other sites were consistent across years. Among children aged 4 years, ASD prevalence was higher in 2014 than in 2010 in New Jersey (PR: 1.4) but not in Arizona or Missouri (Figure 2; Supplemental Table 4, https://stacks.cdc.gov/view/cdc/76016). In Wisconsin, ASD prevalence was higher in 2012 and 2014 than in 2010. Among children aged 8 years living in the Early ADDM Network geographical areas, ASD prevalence was higher in 2014 than in 2010 in New Jersey (PR: 1.3) but not in the other sites.

In Missouri and Wisconsin, ASD prevalence was higher among children aged 8 years than among those aged 4 years in all 3 years (Supplemental Table 4, https://stacks.cdc.gov/view/cdc/76016). In Arizona, ASD prevalence was higher among children aged 8 years than among those aged 4 years in 2012 only, and in New Jersey, no differences by age were found.

### ASD Prevalence Using DSM-IV-TR and DSM-5 Case Definitions

A revised ADDM Network ASD surveillance case definition was developed for the 2014 surveillance year to provide ASD prevalence estimates based on the updated DSM-5 diagnostic criteria published in 2013. All sites reviewed children’s records in the Early ADDM Network by both surveillance case definitions to evaluate the effect on estimated prevalence because of the change to DSM-5 diagnostic criteria. Among children aged 4 years in the Early ADDM Network in 2014, the prevalence of ASD using the DSM-5 surveillance case definition was 14.1 compared with 17.0 for DSM-IV-TR (DSM-IV-TR-to-DSM-5 PR: 1.2) (Table 7). Among 1,237 children who met the surveillance case definition for either DSM-IV-TR or DSM-5, 974 (78.7%) met both case definitions, 234 (18.9%) met the DSM-IV-TR but not the DSM-5 case definition, and 29 (2.3%) met the DSM-5 but not the DSM-IV-TR case definition.

**TABLE 7:** Number and prevalence* of children aged 4 years meeting DSM-IV-TR or DSM-5 autism spectrum disorder case definition — Autism and Developmental Disabilities Monitoring Network, seven sites, United States, 2014

Site and record source	DSM-IV-TR		DSM-5		Prevalence ratio (95% CI),^†^ DSM-IV-TR to DSM-5
	No.	Prevalence (95% CI)	No.	Prevalence (95% CI)	
**Health care and education**					
Arizona	130	13.5 (11.3–16.0)	102	10.6 (8.6–12.9)	1.3 (1.0–1.7)
Colorado	113	13.4 (11.0–16.1)	93	11.0 (8.9–13.5)	1.2 (0.9–1.6)
New Jersey^§^	514	28.4 (26.0–30.9)	406	22.4 (20.3–24.7)	1.3 (1.1–1.4)
North Carolina^§^	231	15.5 (13.6–17.6)	204	13.7 (11.9–15.7)	1.1 (0.9–1.4)
**Health care only**					
Missouri	112	9.6 (7.9–11.6)	105	9.0 (7.4–10.9)	1.1 (0.8–1.4)
Wisconsin^§^	108	13.2 (10.8–15.9)	93	11.3 (9.1–13.9)	1.2 (0.9–1.5)
**Total**	**1,208**	**17.0 (16.1–18.0)**	**1,003**	**14.1 (13.3–15.1)**	**1.2 (1.1–1.3)**

**Abbreviations:** CI = confidence interval; DSM-IV-TR = *Diagnostic and Statistical Manual of Mental Disorders, 4th Edition, Text Revision;* DSM-5 = *Diagnostic and Statistical Manual of Mental Disorder, Fifth Edition*.

* Prevalence per 1,000 children aged 4 years living in the surveillance areas according to the vintage 2016 postcensal bridged-race population estimates for 2014 (http://www.cdc.gov/nchs).

^†^ Results for PRs considered statistically significant when the CI excludes the null value (PR = 1.0).

^§^ Site also reviewed records from early intervention sources.

## Discussion

This report provides data on ASD prevalence among children aged 4 years using ADDM surveillance methods across several sites participating in the Early ADDM Network during 2010, 2012, and 2014. Among these children aged 4 years, overall estimated ASD prevalence was 13.4 per 1,000 in 2010, 15.3 in 2012, and 17.0 in 2014. ASD prevalence was higher among boys than girls. Across all sites and surveillance years, few differences in ASD prevalence were found by race/ethnicity among children aged 4 years, and those that were identified occurred in 2010 but not in later years. In the four sites that participated in Early ADDM Network surveillance in all 3 years, ASD prevalence among children aged 4 years was approximately 40% higher in New Jersey in 2014 than in 2010 and similar across the years in Arizona and Missouri. In Wisconsin, ASD prevalence was significantly higher in 2014 than in 2010. However, the availability of early intervention records in 2014 but not in earlier years might have influenced the prevalence estimates for that year, even though prevalence was similar in 2012 when early intervention records were not reviewed.

The overall prevalence estimate using a DSM-IV-TR case definition was approximately 20% higher than the prevalence estimate based on DSM-5 criteria. Meeting the DSM-5 surveillance case definition required either documentation of the more extensive behavioral criteria required for a DSM-5 diagnosis or an ASD diagnosis by a community provider, and preschool-aged children might have had fewer comprehensive evaluations containing behavioral information and been less likely to have a diagnosis. For the 2016 surveillance year, all ADDM Network surveillance sites will use the DSM-5 case definition, and trends in the prevalence of ASD among children aged 4 years and 8 years will be monitored according to this surveillance case definition.

The estimated ASD prevalence in sites that reviewed both education and health care records was 60%–80% higher than the estimated ASD prevalence among sites that reviewed only health care records. Although ASD prevalence varied even among sites that reviewed education records, the total prevalence among these sites (15.9, 17.4, and 19.3 per 1,000 children aged 4 years, respectively, for 2010–2014) is likely a more sensitive estimate of ASD prevalence among children aged 4 years, suggesting that the overall estimated ASD prevalence in the Early ADDM Network would have been higher had all sites had access to education records. Early intervention records also are an important source of information, particularly for tracking the age at earliest evaluation. For example, the percentage of children evaluated by age 36 months in Wisconsin was higher when early intervention records were included for 2014 but not for earlier years. Together, these findings suggest that early intervention and public education systems are a critical community resource for the evaluation of preschool-aged children who exhibit social, communication, and behavioral impairments. Lack of access to early intervention and education records, combined with indications from earlier reports (*10*–*15*) that many children with ASD are not evaluated until after age 4 years, suggests that the estimate of ASD prevalence among children aged 4 years might be an underestimate of the actual ASD prevalence in this birth cohort.

### Other Studies of ASD Prevalence

Population-based data on the prevalence of ASD in preschool-aged children are limited, and various case ascertainment methods have been used; nevertheless, studies indicate that the prevalence of ASD in this age group has been higher in recent years. In 1996, estimated ASD prevalence among children aged 4 years in MADDSP was 3.1 per 1,000 (95% CI: 2.6–3.7), and the estimated prevalence per 1,000 children aged 8 years was 4.7 (95% CI: 4.0–5.5) (*16*). A study using similar methods conducted in Brick Township, New Jersey, reported an estimated ASD prevalence of 7.8 per 1,000 children aged 3–5 years (95% CI: 5.1–11.3) in 1998 (*30*). A study from South Carolina in 2006 using MADDSP methods found an ASD prevalence of 8.0 per 1,000 children aged 4 years (*31*). A population-based study in the United Kingdom during 1998–1999 that used a multistage screening and diagnosis methodology to identify children with PDD reported a prevalence estimate of 6.3 per 1,000 children aged 3.5–6.5 years (*32*). Another study using the same methods that was conducted several years later in a subsequent birth cohort reported a prevalence estimate of 5.9 per 1,000 children aged 4–6 years (*33*). Approximately 10 years later, a report from the 2007 National Survey of Children’s Health (NSCH) described estimated ASD prevalence by parent or caregiver report to be 8.5 per 1,000 children aged 3–5 years (95% CI: 6.0–12.0), compared with 13.2 per 1,000 children aged 6–8 years (95% CI: 9.6–18.3) (*6*,*34*). Most recently, the 2016 NSCH reported ASD prevalence estimates of 19.7 per 1,000 children aged 3–5 years, 26.1 per 1,000 children aged 6–11 years, and 26.5 per 1,000 children aged 12–17 years (*9*). The most recent data from the National Health Interview Survey showed a prevalence estimate (based on parent or caregiver report) of 22.3 per 1,000 children aged 3–7 years in 2016, which was lower than the prevalence estimate among children aged 8–12 years (28.8) (*34*).

In addition to the findings among preschool-aged children, studies using different surveillance methods also have identified higher ASD prevalence among children in recent years (*5*–*15*,*35*). Several studies highlight changes in community practice for recognizing and diagnosing ASD in children with developmental concerns, as well as expansion of the diagnostic criteria for ASD during 1987–2013 to include children with fewer or more mild symptoms, as factors contributing to the higher prevalence (*36*–*39*). Although assessing whether ASD prevalence trends are, in part, associated with changes in etiologic risk is not possible with ADDM Network data, the heterogeneity of Early ADDM Network prevalence estimates across study sites, even among sites that reviewed both education and health care records, supports the hypothesis that differences in evaluation, diagnostic, and service practices affect measured prevalence. Previous data from the ADDM Network indicate a lower proportion of children with ASD with co-occurring intellectual disabilities (*10*–*15*) over time, consistent with improvements in the identification of children who have milder ASD. In addition, changes in the availability of services for children with ASD through insurance mandates (*40*), willingness of parents and providers to consider an ASD diagnosis, and greater awareness of and concern regarding ASD might contribute to the higher prevalence.

### Early Identification of and Intervention for ASD

The American Academy of Pediatrics prioritized the early identification of ASD through its recommendation for universal ASD screening during pediatric preventive care visits at ages 18 and 24 months (*22*) and by the U.S. Department of Health and Human Services through the Healthy People 2020 goal to increase the proportion of children with ASD who receive their first evaluation by age 36 months. Evidence linking early treatment for ASD with improved outcomes (*18*–*21*,*41*) implies that an absence or a delay in ASD identification could delay interventions and initiation of special services. Identifying the need for special services before school entry to minimize educational disruption and optimize educational outcomes might be especially important.

In this report, across all sites and surveillance years, the median age at first known ASD evaluation among children aged 4 years with ASD ranged from 23 to 37 months, and 48.8% to 88.9% received their first ASD evaluation by age 36 months. The percentage of children with an ASD diagnosis varied widely by study site, ranging from 43.0% to 86.5%, with sites that reviewed only health care records generally reporting a greater percentage of children with an ASD diagnosis. This is not unexpected because other sites include children based wholly or partly on review of education records, which might not contain a formal ASD diagnosis. Among sites with data from all surveillance years and consistent data sources, the age at first evaluation was stable from 2010 to 2014 in Arizona and Missouri. In New Jersey, the age at first evaluation increased from 2010 to 2014. The Wisconsin site gained access to records from early intervention services for children aged <3 years for the 2014 surveillance year, which likely contributed to detecting a greater number of children with a first evaluation by age 36 months. Age at first evaluation might be easier to lower than age at diagnosis because diagnosing ASD in young children is challenging, which might be related to the prodromal nature of autism’s phenotypic onset that has recently become apparent through longitudinal studies of infant siblings at high risk for autism (*42*). However, greater awareness of ASD might result in more children being identified, including those with symptoms that do not fully manifest until the child is close to school age, increasing prevalence while also increasing the age of identification. Prevalence was higher among children aged 8 years than among those aged 4 years in some sites, which might reflect the identification of children with milder symptoms later in development or on school entry; this is supported by the difference in frequency of co-occurring intellectual disabilities between children aged 4 and 8 years. 

Efforts to identify developmental concerns as early as possible and decrease the age at first evaluation for all children with ASD are warranted. As recommended by the American Academy of Pediatrics, universal screening might identify children who need a comprehensive evaluation for ASD, even in the absence of previous developmental concerns or co-occurring intellectual disabilities, and improved tools for discerning the signs of ASD among the range of typical childhood behaviors might aid efforts to identify children earlier. Public health campaigns such as *Learn the Signs. Act Early.* (https://www.cdc.gov/ncbddd/actearly/index.html) provide informational materials for parents, providers, and community members aimed at improving awareness of developmental milestones and increasing early identification of developmental delays so that children can receive appropriate services and treatments as early as possible.

No significant trends were found in the percentage of children with a documented ASD diagnosis or in the age at earliest known diagnosis. Children with an early evaluation can begin to receive behavioral and developmental services and interventions even if a formal ASD diagnosis is not made at that time. However, a formal diagnosis might be necessary to receive certain ASD services; therefore, the 35%–40% of children who met the ASD surveillance case definition but did not have a documented ASD diagnosis might not be eligible for services that depend on an ASD diagnosis.

## Limitations

This report is subject to several limitations. First, because these ASD prevalence estimates are based on a record review, with no clinical examination, Early ADDM Network data reflect the information available in the source records. The amount and quality of the data determine the potential for a child to meet the ASD surveillance case definition and the extent to which they can be used to describe the characteristics of the identified population. Some children with ASD might not have been included because their records were incomplete or not available or they had not come to the attention of schools or clinical providers, which might have resulted in an underestimate of the ASD prevalence. Second, the types of source records varied across surveillance sites, and the lack of availability of education or early intervention records at some sites might have led to an underestimate of ASD prevalence among children aged 4 years in those sites and consequently for the Early ADDM Network overall. Third, early diagnoses of ASD might change if another diagnosis is determined to better account for a child’s signs and symptoms (*6*,*43*,*44*), potentially affecting the specificity of records-based surveillance. However, the ADDM Network clinician review process allows clinicians to change the ASD surveillance case status, even if the child has a previous ASD diagnosis, which helps decrease potential overestimates. Fourth, the availability of early intervention records in Wisconsin for 2014 but not for earlier years prevented the interpretation of changes in prevalence as well the age at earliest developmental evaluation and ASD diagnosis for that site. Fifth, measurement of intellectual ability in preschool-aged children is less reliable than measurement among school-aged children (*26*,*27*), preventing more specific classification of intellectual ability among children with ASD other than the presence or absence of intellectual disability. Sixth, data on intellectual ability were not available for all children, and the distribution of intellectual ability among the children with these data might not be generalizable to all children with ASD in the Early ADDM Network if the data on intellectual ability are not randomly missing. For example, children without a cognitive test score might not have been tested because their intellectual ability was clearly in the average to above-average range, thus overestimating the proportion of children with ASD and co-occurring intellectual disabilities. Seventh, the surveillance sites were selected through a competitive process and were not selected to be representative of children aged 4 years either in the United States or in the entire state in which surveillance occurred. Therefore, the estimated prevalence of ASD is limited to the surveillance areas. Finally, analyses of trends were limited to three sites with data and consistent data sources for all 3 surveillance years, and within sites, data were sparse for certain race/ethnicity groups. In addition, patterns of ASD prevalence and characteristics varied by site; therefore, in some cases, data could not be combined, limiting the statistical power.

### Estimating ASD Prevalence Using Surveillance Data 

Surveillance data from the Early ADDM Network provides 1) population-based ascertainment of ASD using multiple community data sources, including education and early intervention records for some sites; 2) inclusion of children with documentation of behaviors consistent with ASD but without a documented ASD diagnosis; 3) data on intellectual disability based on standardized tests of intellectual ability; and 4) collection of information on the age at first comprehensive evaluation and ASD diagnosis, when present, that provide information on early identification of children with ASD. The record review method allows population-based estimates of ASD prevalence to be generated cost-effectively. Obtaining data from multiple community sources helps to improve the sensitivity of the surveillance system; education and early intervention records provide important information on services and early identification of children with ASD. The inclusion of children without a documented ASD diagnosis allows the surveillance system to identify children who might have less access to the health care system, such as children who receive evaluation services only in school where a formal ASD diagnosis might not be provided. Although the estimates are not representative of the United States or the state where each site was located, surveillance conducted in smaller areas close to evaluation and diagnostic centers might provide a more valid prevalence estimate than for larger areas where services might be lacking. Finally, the validity of the surveillance system compared with clinical examination of children has been assessed among children aged 8 years in a study using MADDSP data, which concluded that the ADDM method was unlikely to overestimate ASD prevalence, although some cases might be missed that would be identified an in-person evaluation using gold standard diagnostic instruments (*45*).

## Conclusion

ASD surveillance among children aged 4 years provides information on progress made toward early identification goals and informs providers, particularly public schools, of upcoming service needs. ASD prevalence was stable in some sites participating in the Early ADDM Network and was higher in 2014 than 2010 in one site; the higher prevalence might reflect improved identification of children with ASD by community providers. Lack of access to education records in some sites might have limited the sensitivity of records-based surveillance in those sites. However, variations in prevalence did not always align with access to data sources, and differences in evaluation and diagnostic services among different areas might account for some differences in findings across surveillance sites. This suggests that opportunities for improvements in services might exist based on successful programs implemented in specific areas. Continuing improvements in providing developmental evaluations to children as soon as developmental concerns are identified might result in earlier ASD diagnoses and earlier receipt of services, which might improve developmental outcomes. No treatment for ASD is available, although interventions might maximize each child’s ability to function and participate in the community (*18*–*21*,*41*).

## Appendix

### Detailed Method for Estimating Surveillance Area Population Size of Partial Counties

For 2010, the number of children aged 4 years by sex and race/ethnicity was obtained for each census tract in the county from the 2010 decennial census counts. Next, each census tract was matched to the school district or districts to which it was fully or partially allocated, using the MABLE/*Geocorr12*: Geographic Correspondence Engine provided by the Missouri Census Data Center (http://mcdc.missouri.edu). A list of excluded or partially excluded census tracts was compiled, and the number of children aged 4 years living in these census tracts was subtracted from the overall and sex- and race-specific total numbers of children in the county. For census tracts that were partially allocated to a school district, weighting was based on the 2010 census population of the county. Finally, population counts of children aged 4 years in each specific census race/ethnicity category (white, black, American Indian/Alaska Native, Asian/Pacific Islander, other race/ethnicity, multiracial, and Hispanic) were adjusted to the distribution of the National Center on Health Statistics (NCHS) bridged-race category counts for the county, thereby incorporating children categorized in the census counts as multiracial or other race into the bridged-race categories reported by NCHS (white, black, Hispanic, American Indian/Alaska Native, and Asian/Pacific Islander). The same methods were used to estimate the prevalence of ASD among children aged 8 years living in the Early ADDM Network surveillance area.

For the nondecennial census years 2012 and 2014, denominators for sites that covered less than a full county were estimated by using school enrollment counts for the appropriate grades in the covered area and applying the distribution of these counts to the county-level bridged-race postcensal population estimates from NCHS (http://www.cdc.gov/nchs).

## References

[1] American Psychiatric Association. Diagnostic and statistical manual of mental disorders. 5th ed. Arlington, VA: American Psychiatric Association; 2013.

[2] Croen LA, Grether JK, Hoogstrate J, Selvin S. The changing prevalence of autism in California. J Autism Dev Disord 2002;32:207–15. 10.1023/A:1015453830880 12108622

[3] Newschaffer CJ, Falb MD, Gurney JG. National autism prevalence trends from United States special education data. Pediatrics 2005;115:e277–82. 10.1542/peds.2004-1958 15741352

[4] California Department of Developmental Services. Autistic spectrum disorders: changes in the California caseload, an update: June 1987–June 2007. Sacramento, CA: California Health and Human Services Agency, Department of Developmental Services; 2007.

[5] Blumberg SJ, Bramlett MD, Kogan MD, Schieve LA, Jones JR, Lu MC. Changes in prevalence of parent-reported autism spectrum disorder in school-aged U.S. children: 2007 to 2011–2012. Natl Health Stat Rep 2013;65:1–11. 24988818

[6] Kogan MD, Blumberg SJ, Schieve LA, Prevalence of parent-reported diagnosis of autism spectrum disorder among children in the US, 2007. Pediatrics 2009;124:1395–403. 10.1542/peds.2009-1522 19805460

[7] Schieve LA, Rice C, Yeargin-Allsopp M, Parent-reported prevalence of autism spectrum disorders in U.S.-born children: an assessment of changes within birth cohorts from the 2003 to the 2007 National Survey of Children’s Health. Matern Child Health J 2012;16(Suppl 1):S151–7. 10.1007/s10995-012-1004-0 22476793

[8] Zablotsky B, Black LI, Maenner MJ, Schieve LA, Blumberg SJ. Estimated prevalence of autism and other developmental disabilities following questionnaire changes in the 2014 National Health Interview Survey. Natl Health Stat Report 2015;87:1–20. 26632847

[9] Kogan MD, Vladutiu CJ, Schieve LA, The prevalence of parent-reported autism spectrum disorder among U.S. children. Pediatrics 2018;142:e20174161. 10.1542/peds.2017-4161 30478241 PMC6317762

[10] Autism and Developmental Disabilities Monitoring Network Surveillance Year 2002 Principal Investigators. Prevalence of autism spectrum disorders—Autism and Developmental Disabilities Monitoring Network, four sites, United States, 2002. MMWR Surveill Summ 2007;56(No. SS-1). 17287715

[11] Autism and Developmental Disabilities Monitoring Network Surveillance Year 2006 Principal Investigators. Prevalence of autism spectrum disorders—Autism and Developmental Disabilities Monitoring Network, United States, 2006. MMWR Surveill Summ 2009;58(No. SS-10). 20023608

[12] Autism and Developmental Disabilities Monitoring Network Surveillance Year 2008 Principal Investigators. Prevalence of autism spectrum disorders—Autism and Developmental Disabilities Monitoring Network—four sites, United States, 2008. MMWR Surveill Summ 2012;61(No. SS-3). 22456193

[13] Baio J, Wiggins L, Christensen DL, Prevalence of autism spectrum disorder among children aged 8 years—Autism and Developmental Disabilities Monitoring Network, 11 sites, United States, 2014. MMWR Surveill Summ 2018;67:1–23. 10.15585/mmwr.ss6706a1 29701730 PMC5919599

[14] Christensen DL, Braun KVN, Baio J, Prevalence of autism spectrum disorder among children aged 8 years—Autism and Developmental Disabilities Monitoring Network, 11 sites, United States, 2012. MMWR Surveill Summ 2018;65(No. SS-13). 10.15585/mmwr.ss6513a1 PMC6237390 30439868

[15] Autism and Developmental Disabilities Monitoring Network Surveillance Year 2010 Principal Investigators. Prevalence of autism spectrum disorder among children aged 8 years—Autism and Developmental Disabilities Monitoring Network, 11 sites, United States, 2010. MMWR Surveill Summ 2014;63(No. SS-2). 24670961

[16] Yeargin-Allsopp M, Rice C, Karapurkar T, Doernberg N, Boyle C, Murphy C. Prevalence of autism in a U.S. metropolitan area. JAMA 2003;289:49–55. 10.1001/jama.289.1.49 12503976

[17] Autism and Developmental Disabilities Monitoring Network Surveillance Year 2000 Principal Investigators. Prevalence of autism spectrum disorders—autism and developmental disabilities monitoring network, six sites, United States, 2000. MMWR Surveill Summ 2007;56:1–11. 17287714

[18] Dawson G, Rogers S, Munson J, Randomized, controlled trial of an intervention for toddlers with autism: the Early Start Denver Model. Pediatrics 2010;125:e17–23. 10.1542/peds.2009-0958 19948568 PMC4951085

[19] Eapen V, Crnčec R, Walter A. Clinical outcomes of an early intervention program for preschool children with Autism Spectrum Disorder in a community group setting. BMC Pediatr 2013;13:3. 10.1186/1471-2431-13-3 23294523 PMC3631131

[20] Reichow B, Barton EE, Boyd BA, Hume K. Early intensive behavioral intervention (EIBI) for young children with autism spectrum disorders (ASD). Cochrane Database Syst Rev 2012;10:CD009260. 10.1002/14651858.CD009260.pub2 23076956

[21] Rogers SJ, Estes A, Lord C, Effects of a brief Early Start Denver model (ESDM)-based parent intervention on toddlers at risk for autism spectrum disorders: a randomized controlled trial. J Am Acad Child Adolesc Psychiatry 2012;51:1052–65. 10.1016/j.jaac.2012.08.003 23021480 PMC3487718

[22] Johnson CP, Myers SM; American Academy of Pediatrics Council on Children With Disabilities. Identification and evaluation of children with autism spectrum disorders. Pediatrics 2007;120:1183–215. 10.1542/peds.2007-2361 17967920

[23] Rice CE, Baio J, Van Naarden Braun K, Doernberg N, Meaney FJ, Kirby RS; ADDM Network. A public health collaboration for the surveillance of autism spectrum disorders. Paediatr Perinat Epidemiol 2007;21:179–90. 10.1111/j.1365-3016.2007.00801.x 17302648

[24] US Department of Health and Human Services. Code of Federal Regulations. Title 45. Public Welfare CFR 46. Washington, DC: US Department of Health and Human Services; 2010.

[25] American Psychiatric Association. Diagnostic and statistical manual of mental disorders. 4th ed, Text Revision. Washington, DC: American Psychiatric Association; 2000.

[26] Lord C, Schopler E. The role of age at assessment, developmental level, and test in the stability of intelligence scores in young autistic children. J Autism Dev Disord 1989;19:483–99. 10.1007/BF02212853 2606880

[27] Sattler J. Assessment of children’s intelligence and special abilities. Boston, MA: Allyn & Bacon; 1982.

[28] Christensen DL, Bilder DA, Zahorodny W, Prevalence and characteristics of autism spectrum disorder among 4-year-old children in the Autism and Developmental Disabilities Monitoring Network. J Dev Behav Pediatr 2016;37:1–8. 10.1097/DBP.0000000000000235 26651088

[29] US Census Bureau. Census summary file 1: Tables PCT12H–PCT12O. Washington, DC: US Census Bureau; 2010.

[30] Bertrand J, Mars A, Boyle C, Bove F, Yeargin-Allsopp M, Decoufle P. Prevalence of autism in a United States population: the Brick Township, New Jersey, investigation. Pediatrics 2001;108:1155–61. 10.1542/peds.108.5.1155 11694696

[31] Nicholas JS, Carpenter LA, King LB, Jenner W, Charles JM. Autism spectrum disorders in preschool-aged children: prevalence and comparison to a school-aged population. Ann Epidemiol 2009;19:808–14. 10.1016/j.annepidem.2009.04.005 19541501

[32] Chakrabarti S, Fombonne E. Pervasive developmental disorders in preschool children. JAMA 2001;285:3093–9. 10.1001/jama.285.24.3093 11427137

[33] Chakrabarti S, Fombonne E. Pervasive developmental disorders in preschool children: confirmation of high prevalence. Am J Psychiatry 2005;162:1133–41. 10.1176/appi.ajp.162.6.1133 15930062

[34] Zablotsky B, Black LI, Blumberg SJ. Estimated prevalence of children with diagnosed developmental disabilities in the United States, 2014–2016. NCHS Data Brief, No. 291. Hyattsville, MD: CDC, National Center for Health Statistics; 2017;291:1–8. 29235982

[35] Boyle CA, Boulet S, Schieve LA, Trends in the prevalence of developmental disabilities in U.S. children, 1997–2008. Pediatrics 2011;127:1034–42. 10.1542/peds.2010-2989 21606152

[36] Hansen SN, Schendel DE, Parner ET. Explaining the increase in the prevalence of autism spectrum disorders: the proportion attributable to changes in reporting practices. JAMA Pediatr 2015;169:56–62. 10.1001/jamapediatrics.2014.1893 25365033

[37] Hertz-Picciotto I, Delwiche L. The rise in autism and the role of age at diagnosis. Epidemiology 2009;20:84–90. 10.1097/EDE.0b013e3181902d15 19234401 PMC4113600

[38] Lundström S, Reichenberg A, Anckarsäter H, Lichtenstein P, Gillberg C. Autism phenotype versus registered diagnosis in Swedish children: prevalence trends over 10 years in general population samples. BMJ 2015;350:h1961. 10.1136/bmj.h1961 25922345 PMC4413835

[39] Nassar N, Dixon G, Bourke J, Autism spectrum disorders in young children: effect of changes in diagnostic practices. Int J Epidemiol 2009;38:1245–54. 10.1093/ije/dyp260 19737795

[40] Mandell DS, Barry CL, Marcus SC, Effects of autism spectrum disorder insurance mandates on the treated prevalence of autism spectrum disorder. JAMA Pediatr 2016;170:887–93. 10.1001/jamapediatrics.2016.1049 27399053

[41] Dawson G, Jones EJ, Merkle K, Early behavioral intervention is associated with normalized brain activity in young children with autism. J Am Acad Child Adolesc Psychiatry 2012;51:1150–9. 10.1016/j.jaac.2012.08.018 23101741 PMC3607427

[42] Piven J, Elison JT, Zylka MJ. Toward a conceptual framework for early brain and behavior development in autism. Mol Psychiatry 2017;22:1385–94. 10.1038/mp.2017.131 28937691 PMC5621737

[43] Pringle B, Colpe LJ, Blumberg SJ, Avila RM, Kogan MD. Diagnostic history and treatment of school-aged children with autism spectrum disorder and special health care needs. NCHS Data Brief 2012;97:1–8. 23050521

[44] Blumberg SJ, Zablotsky B, Avila RM, Colpe LJ, Pringle BA, Kogan MD. Diagnosis lost: differences between children who had and who currently have an autism spectrum disorder diagnosis. Autism 2016;20:783–95. 10.1177/1362361315607724 26489772 PMC4838550

[45] Avchen RN, Wiggins LD, Devine O, Evaluation of a records-review surveillance system used to determine the prevalence of autism spectrum disorders. J Autism Dev Disord 2011;41:227–36. 10.1007/s10803-010-1050-7 20568003

